# Integrated 16S rRNA sequencing and metagenomics insights into microbial dysbiosis and distinct virulence factors in inflammatory bowel disease

**DOI:** 10.3389/fmicb.2024.1375804

**Published:** 2024-03-25

**Authors:** Haijing Wang, Yuanjun Wang, Libin Yang, Jiawen Feng, Shou Tian, Lingyan Chen, Wei Huang, Jia Liu, Xiaojin Wang

**Affiliations:** ^1^Medical College of Qinghai University, Xining, China; ^2^Qinghai University Affiliated Hospital, Xining, China; ^3^Ningxia Hui Autonomous Region People's Hospital, Yinchuan, China; ^4^Qinghai Provincial Traditional Chinese Medicine Hospital, Xining, China

**Keywords:** 16S rRNA, metagenome, inflammatory bowel disease, Crohn's disease, ulcerative colitis, case-control study

## Abstract

**Introduction:**

The escalation of urbanization correlates with rising rates of inflammatory bowel disease (IBD), necessitating research into new etiological factors. This study aims to elucidate the gut microbiota profiles in IBD patients and compare them with healthy controls in a western city of China.

**Methods:**

We conducted a multicenter case-control study from the end of 2020, using 16S rRNA gene sequencing (*n* = 36) and metagenomic sequencing (*n* = 12) to analyze the gut microbiota of newly diagnosed IBD patients, including those with Crohn's disease (CD) and ulcerative colitis (UC).

**Results:**

Our results demonstrated a significant enrichment of the phylum Proteobacteria, particularly the genus *Escherichia-Shigella*, in CD patients. Conversely, the genus *Enterococcus* was markedly increased in UC patients. The core gut microbiota, such as the *Christensenellaceae R-7* group, *Fusicatenibacter*, and *Holdemanella*, were primarily identified in healthy subjects. Additionally, significant interactions between the microbiome and virulence factors were observed.

**Discussion:**

The findings suggest that oxidative stress may play a pivotal role in the pathology of IBD. This study contributes to the growing dialogue about the impact of gut microbiota on the development of IBD and its variations across different geographies, highlighting potential avenues for further research.

## 1 Introduction

Inflammatory bowel disease (IBD) represents a group of chronic inflammatory disorders of the gastrointestinal tract, primarily encompassing Crohn's disease (CD) and ulcerative colitis (UC). IBD is a major public health burden due to its substantially increased prevalence in many regions (GBD 2017 Inflammatory Bowel Disease Collaborators, 2020). Traditionally, the incidence and prevalence of IBD were lower in Asian populations compared to western populations (Kaplan and Windsor, 2021). However, the latest data revealed a significant shift, particularly in newly industrialized countries where occurrences are influenced by environmental pressures such as nutrition and lifestyle behaviors, with China and the USA currently reporting the highest numbers of IBD cases worldwide (Park and Cheon, 2021; Wang et al., 2023). Due to the speed of industrialization and a fast-growing aging population, the burden of IBD in China has steadily increased and is expected to increase further by 2030 (Shao et al., 2022; Ma et al., 2023). Although the prevalence and incidence in the Western region of China lag behind those in the Eastern region (Yang et al., 2022), the implications of rapid urbanization should not be ignored.

Although the cause of IBD remains unknown, emerging evidence increasingly highlights the critical role of the microbiome in IBD patients. A cohort study and Mendelian randomization analyses have further confirmed the causal relationship between individual taxa and CD (Raygoza Garay et al., 2023; Li et al., 2024). The advent of high-throughput sequencing technologies, particularly 16S rRNA gene and metagenome sequencing, have revolutionized our comprehension of the intricate microbial communities in the human gut and their contributions to the onset and progression of IBD (Basha et al., 2023; Ning et al., 2023). Gut microbiota's integral connection to human physiology and diet, alongside the interplay between geographical origin and disease, adds complexity to assessing disease-associated alterations in the microbial populations (Rehman et al., 2016; He et al., 2022; Zhang and Gérard, 2022). Given the fact that the Western region exhibits a multi-ethnic composition and distinct dietary patterns characterized by animal husbandry lifestyles (Zhang et al., 2023), the heterogeneity of IBD geographical distribution makes it intriguing to delineate the microbial factor associated with IBD conditions.

The human gastrointestinal tract is a sophisticated and ever-evolving ecosystem, teeming with a diverse consortium of microorganisms, including bacteria and viruses. The intricate trans-kingdom interplay among gut viruses, bacteria, and the mammalian host plays a critical role in influencing both health and disease, and the gut virome is highly heterogeneous across populations (Cao et al., 2022). Recent studies have highlighted the role of the gut virome in the pathophysiology of IBD and its correlation with therapeutic success, indicating that these viruses might serve as novel targets for treatment (Jansen et al., 2023; Massimino et al., 2023). Furthermore, the expansive realm of metagenomics has redirected our focus toward the broader implications of the interactions in this complex community.

Given the limited research on IBD in western China and its distinct dietary patterns, this study aims to utilize 16S rRNA gene and metagenome sequencing to investigate the gut microbiota profiles of IBD patients. The objective of this study is to explore the relationship between microbial community networks and IBD, thereby deepening our understanding of these intricate interactions across diverse geographical areas.

## 2 Materials and methods

### 2.1 Participants and samples

A total of 36 participants were recruited from two hospitals (Qinghai Provincial Traditional Chinese Medicine Hospital and Qinghai Provincial People's Hospital) at Xining in Qinghai Province, China, from 2020. The *de novo* diagnosis of 18 IBD patients was matched by age and gender with 18 healthy individuals (CTL). The CTL group was further differentiated into distinct controls for cases in CD (CD_CTL) and UC (UC_CTL), enabling a more accurate comparative analysis. Patients with CD (*n* = 4) and UC (*n* = 14) were further classified into subtypes using the Montreal classification system, and disease severity was meticulously assessed (Table 1). Demographic characteristics were collected by questionnaire, and biochemical examinations were conducted using the collected fecal samples. A minimum of 2 g of the central part of the fecal sample was collected with a spoon in the gut collection cup; then, the cup was numbered and kept frozen in the laboratory at −80°C within 2 h of collection of the sample. This project was approved by the ethics committee of the Qinghai Provincial Traditional Chinese Medicine Hospital.

**Table 1:** The detail of *de novo* diagnosis of IBD patients.

**Categories**	**Variables**	**Levels**	**CD (*n* = 4)**	**UC (*n* = 14)**
Disease subtype	Subtype A (the age of onset)	A1	0 (0%)	
		A2	1 (25%)	
		A3	3 (75%)	
	Subtype B/P (disease behavior)	B1	2 (50%)	
		B2	0 (0%)	
		B3	2 (50%)	
	Subtype L (disease location)	L1	1 (25%)	
		L2	0 (0%)	
		L3	3 (75%)	
	Subtype E (disease behavior)	E1		0 (0)
		E2		4 (28.57%)
		E3		10 (71.43%)
Severity grading	CD Activity Index (Mean ± SD)		124.30 ± 39.65	
	UC Mayo Score (Mean ± SD)			7.14 ± 2.28

### 2.2 Microbiota 16s rRNA sequencing

Total genomic DNA was extracted from the gut microbiome samples, and the 16S V4-V5 region was amplified with primer pairs 338F (5′-ACTCCTACGGGAGGCAGCAG-3′) and 806R (5′-GGACTACHVGGGTWTCTAAT-3′). Then, the products were purified and quantified to create a library and sequenced by the Illumina MiSeq PE300 platform (Illumina, San Diego, USA). The raw amplicon sequence variants (ASVs) dataset was acquired by the Qiime2 pipeline (version 2020.2), employing the recommended parameters. The taxonomy of ASVs was classified with the Q2-feature-classifier's classify-sklearn tool, setting the confidence threshold at 0.8. We then conducted taxonomy-based filtering to exclude ASVs identified as mitochondria, chloroplasts, or archaea. Additionally, we removed ASVs that were present in <10% of the samples or had a relative abundance below 0.0001%. The rarefaction curve analysis confirmed that the sequencing depth for each sample met the anticipated targets (Supplementary Figure 1A).

### 2.3 Metagenomic sequencing and data analysis

Metagenomic sequencing was performed on samples randomly selected from 16s rRNA sequencing samples with six IBD (three UC and three CD) patients and their six matched healthy counterparts (CTL). DNA was extracted, and libraries were constructed; then paired-end sequencing was executed using an Illumina NovaSeq/Hiseq X Ten platform (Illumina Inc., San Diego, CA, USA) at Majorbio Bio-Pharm Technology Co., Ltd. Adapter sequences and low-quality reads (shorter than 50 bp, with a quality score below 20, or containing ambiguous bases denoted as “N”) were filtered out using fastp (Chen et al., 2018). The remaining high-quality reads were then mapped to the human genome using the Burrows-Wheeler Alignment (BWA) tool l (Li and Durbin, 2009), with any human-aligned reads, and their mate discarded. The MEGAHIT was conducted to assemble the metagenomic data, and representative sequences of the non-redundant gene catalog were aligned to the NR database with an e-value cutoff of 1e-5 using Diamond for taxonomic annotations. A cluster of orthologous groups of the Kyoto Encyclopedia of Genes and Genomes (KEGG) database and the Virulence Factor Database (VFDB; Kanehisa and Goto, 2000; Liu et al., 2022) were also used for functional annotation and further function assignment.

### 2.4 Alpha and beta diversity analysis

Alpha-diversity, represented by the Chao, Sob, and Shannon indexes, was estimated based on the ASV profiles from 16S rRNA sequences and at the species level from metagenomic profiles. The richness of the microbiomes was evaluated using the Sobs and Chao indexes, while the diversity was assessed by the Shannon index. Beta-diversity was visualized through non-metric multidimensional scaling (NMDS), employing a Bray-Curtis dissimilarity matrix estimated from the square-root-transformed and Wisconsin double-standardized ASV table. The beta diversity distance value was calculated using the Bray-Curtis dissimilarity matrix from the metagenomic profile.

### 2.5 Statistical analysis of demographic and microbiome data

Demographic information was expressed as the mean ± standard deviation, unless otherwise indicated, and analyzed using the paired *t*-test or chi-square test for statistical significance. Considering that microbial data were sparse with a non-normal distribution, relative statistics were performed with a non-parametric test, such as the Wilcoxon test. The tableone package was conducted to construct “Table 1” (https://github.com/kaz-yos/tableone). All statistical analyses were conducted using the “stats” package in R. For identifying taxa with the most pronounced differences in abundance and function across groups, we performed linear discriminant analysis effect size (LEfSe) analysis. The co-occurrence network calculated by networkX vividly illustrates the symbiotic relationships between the genus and samples, thereby facilitating an enhanced understanding of the distribution patterns of dominant species across various samples, and is visualized in Cytoscape (v 3.10). The comprehensive Spearman correlation analysis was performed between the microbiome at the genus level (top 50) and various virulence factors (top 50) in metagenome sequencing. We established a correlation coefficient threshold of 0.5, and only associations with a *p*-value of <0.05 were considered statistically significant and were retained for further visualization in Gephi (v 0.1). The visualization of the results was facilitated by the “ggplot2” package (Wickham, 2016) and the “microeco” package (Liu et al., 2021) in R, enabling a comprehensive and detailed graphical representation of our findings.

## 3 Results

### 3.1 Characteristics of participants

We enrolled 36 participants, 24 men and 12 women, aged 46.17 ± 11.48 years, and most of them were of Han ethnicity. IBD patients, including 14 UC and 4 CD patients, were paired with healthy volunteers of the same age and gender. The comparison results revealed no differences between patients and healthy participants (Table 2), indicating that these demographic characteristics were comparable across groups.

**Table 2:** Demographic characteristics of participants (*n* = 36).

	**UC_CTL**	**UC**	***p***	**CD_CTL**	**CD**	***p***
	**(*****n*** = **14)**	**(*****n*** = **14)**		**(*****n*** = **4)**	**(*****n*** = **4)**	
Age [mean (SD)]	46.43 (12.70)	45.64 (12.62)	0.9	48.50 (9.85)	44.75 (6.24)	0.54
Gender (%)						
Men	8 (57.1)	8 (57.1)		4 (100.0)	4 (100.0)	
Women	6 (42.9)	6 (42.9)		0 (0.0)	0 (0.0)	
Ethnic group (%)			0.4			0.15
Han	8 (57.1)	10 (71.4)		1 (25.0)	0 (0.0)	
Tibetans	5 (35.7)	2 (14.3)		2 (50.0)	1 (25.0)	
Others	1 (7.1)	2 (14.3)		1(25.0)	3 (75.0)	
Education (%)			0.7			0.29
< 9 years of schooling	6 (42.9)	5 (35.7)		4 (100.0)	3 (75.0)	
≥9 years of schooling	8 (57.1)	9 (64.3)		0 (0.0)	1 (25.0)	
Marital (%)			0.6			1
Unmarried/widowed/divorced/separated	3 (21.4)	2 (14.3)		0 (0.0)	0 (0.0)	
Married	11 (84.6)	12 (85.7)		4 (100.0)	4 (100.0)	
Occupational labor intensity (%)			0.3			0.31
Light	9 (69.2)	9 (64.3)		1 (25.0)	0 (0.0)	
Moderate	5 (30.8)	3 (21.4)		2 (50.0)	1 (25.0)	
Heavy	0 (0.0)	2 (14.3)		1 (25.0)	3 (75.0)	
BMI [mean (SD)]	23.30 (3.70)	22.88 (3.11)	0.8	25.70 (3.82)	21.52 (3.27)	0.15

### 3.2 Decreased richness and diversity in IBD patients

**Figure 1:**
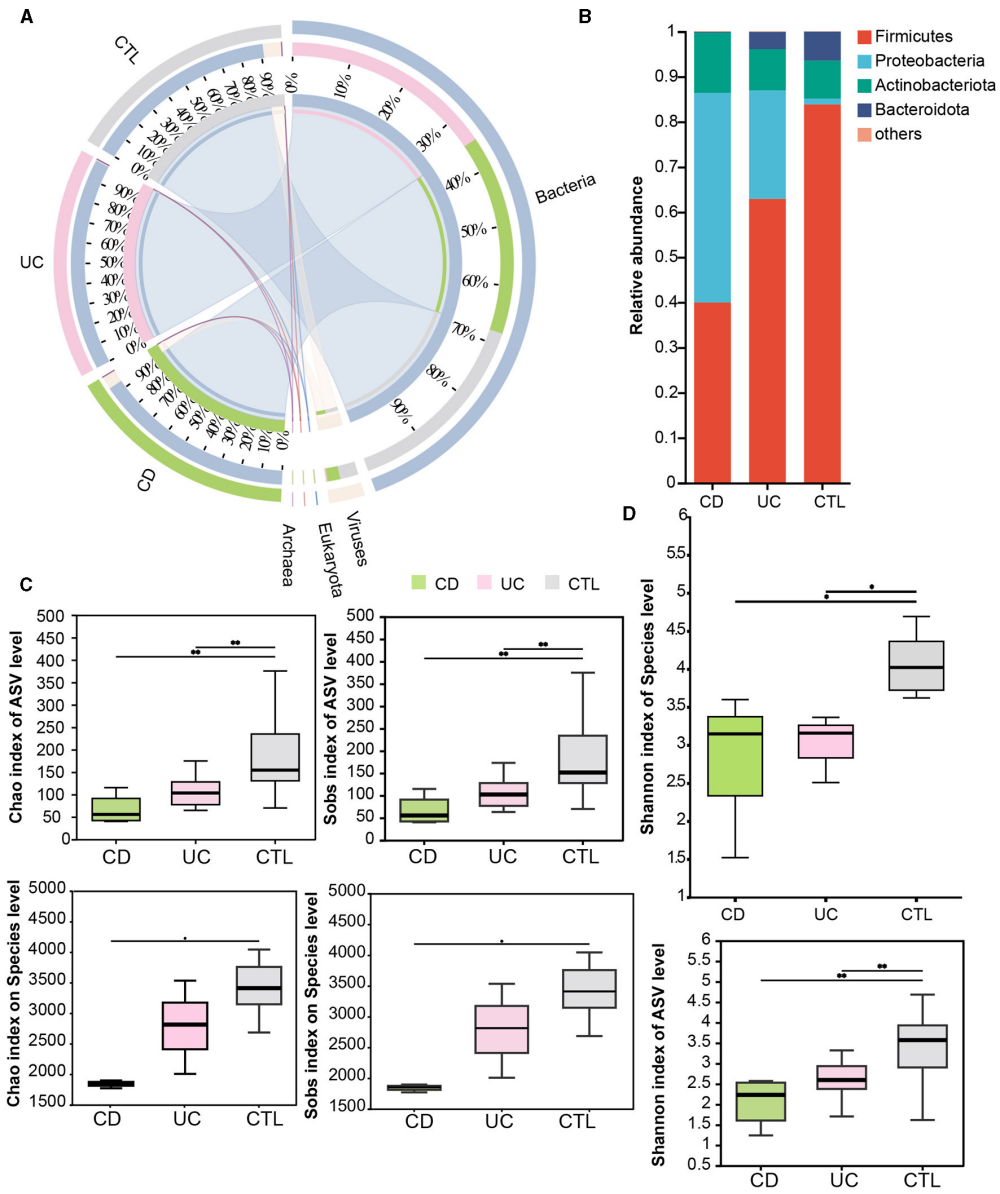
The composition and alpha diversity in participants. **(A)** The circoplot of microbial composition at the domain level. **(B)** The barplot of composition at the phylum level. **(C)** The difference of richness in microbial species. **(D)** The difference of the Shannon index in microbial species. The upper panel shows results from 16S rRNA sequencing and the lower panel from metagenomics analysis both in **(C, D)**. The Wilcoxon test was conducted, and * indicated *p* < 0.05, ** indicated *p* < 0.01. CD means Crohn's Disease patients, UC means Ulcerative Colitis patients, and CTL means healthy participants.

Significant differences in the gut microbiota composition and diversity were observed between patients with IBD and healthy individuals (Supplementary Figure 1B). These variations were characterized using 16S rRNA sequencing to analyze microbial composition and diversity, complemented by metagenomic approaches for an extensive microbial overview. Notably, bacteria emerged as the most abundant microorganisms in the participants, with a marked enrichment of viruses in CD (40%) compared to UC (3%) patients (Figure 1A). Furthermore, 16S rRNA sequencing highlighted substantial alterations in the composition, richness, and diversity of the gut microbiota among IBD patients. At the phylum level, the dominant bacteria included *Firmicutes, Proteobacteria, Actinobacteriota*, and *Bacteroidetes* (Figure 1B). At the genus level, the alterations were equally striking, and *Escherichia-Shigella* was predominant in both CD and UC patients, accounting for 59 and 50% of the microbiota, respectively. Furthermore, *Bifidobacterium* and *Streptococcus* were identified as the sub-dominant bacterial genera in CD (21%) and UC (23%) patients, respectively (Supplementary Figure 1C). The richness and diversity of the bacterial community at the ASV level, as measured by the Sobs and Chao indices, were significantly reduced in both UC and CD patients (Figure 1C). Moreover, the Shannon index, which was expanded at the species level in the metagenomic data (Figure 1D), corroborated with the 16S rRNA findings, indicating a reduction in alpha diversity within the IBD patients.

### 3.3 Changes in beta diversity and the differentially dominant bacteria

**Figure 2:**
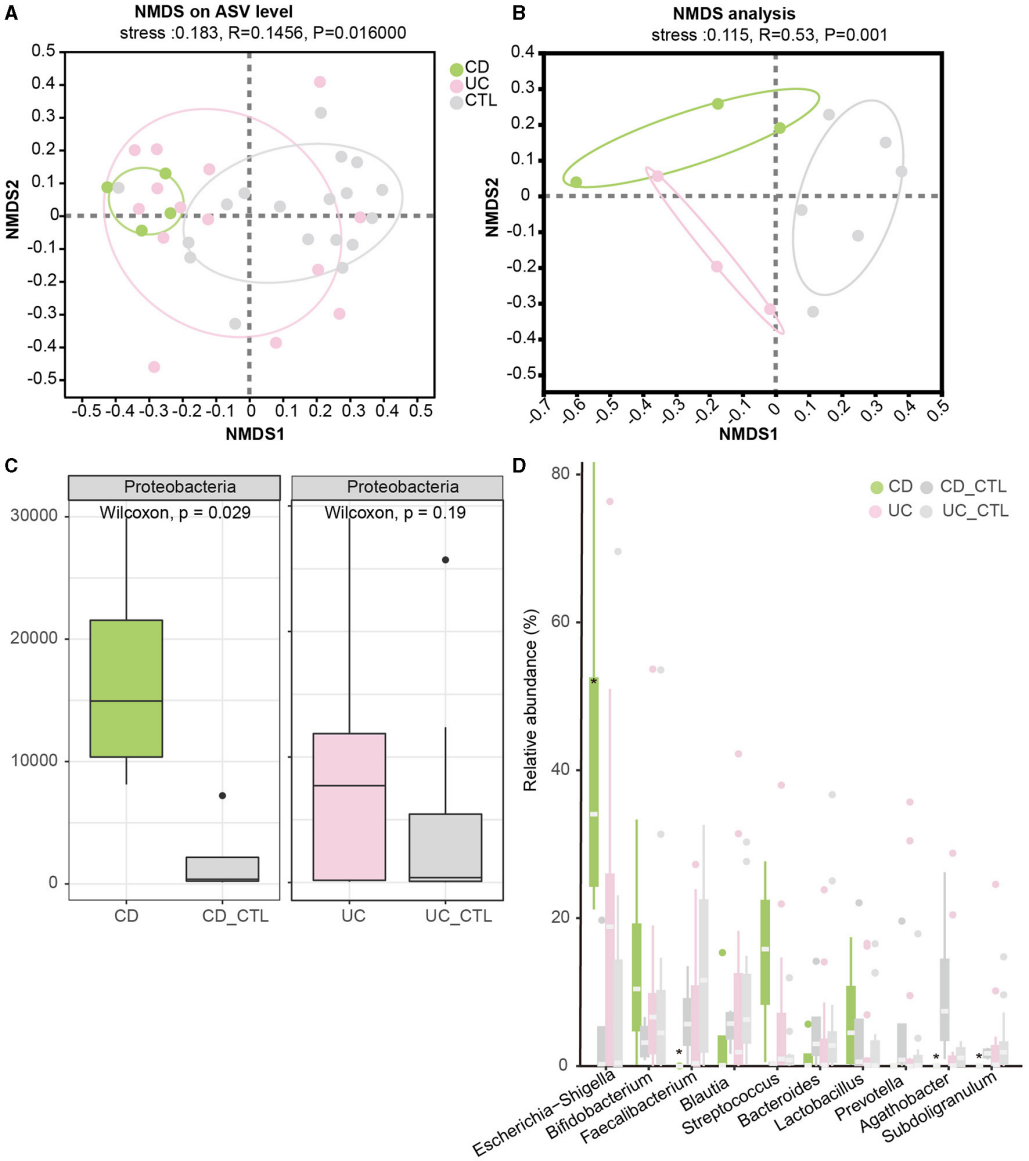
Beta diversity among groups and the significant dominant bacterias. **(A, B)** Depict the NMDS results in 16S rRNA sequencing and metagenome sequencing, respectively. **(C)** The boxplot demonstrates the difference in Proteobacteria. The left panel compares CD patients, while the right panel compares UC patients. **(D)** The boxplot of the top 10 bacteria at the genus level, with * indicating a significant difference (*p* < 0.05). CD_CTL and UC_CTL were healthy controls for CD and UC patients, respectively.

To investigate the difference between IBD patients and healthy participants, the NMDS analysis was conducted to explore beta diversity. The differences of beta diversity in gut microbiota were identified at the ASV level (Figure 2A), with IBD samples primarily distributed in the left region, showing some overlaps between CD and UC patients, whereas healthy participants were located in the right region. The NMDS analysis of the metagenome profile (stress = 0.115) revealed a similar relationship between the sampling sites, indicating that these regions contained distinct bacterial community structures (Figure 2B). To figure out the difference among the dominant bacteria, analyses at both phylum and genus levels were conducted. The phylum Proteobacteria showed a significant increase in IBD patients (Supplementary Figure 1D), while further analysis indicated that this enrichment was specific to CD patients (Figure 2C). In CD patients, at the genus level, *Escherichia-Shigella* significantly increased, while *Faecalibacterium, Agathobacter*, and *Subdoligranulum* significantly decreased (Figure 2D).

### 3.4 The taxonomy biomarkers of IBD patients

**Figure 3:**
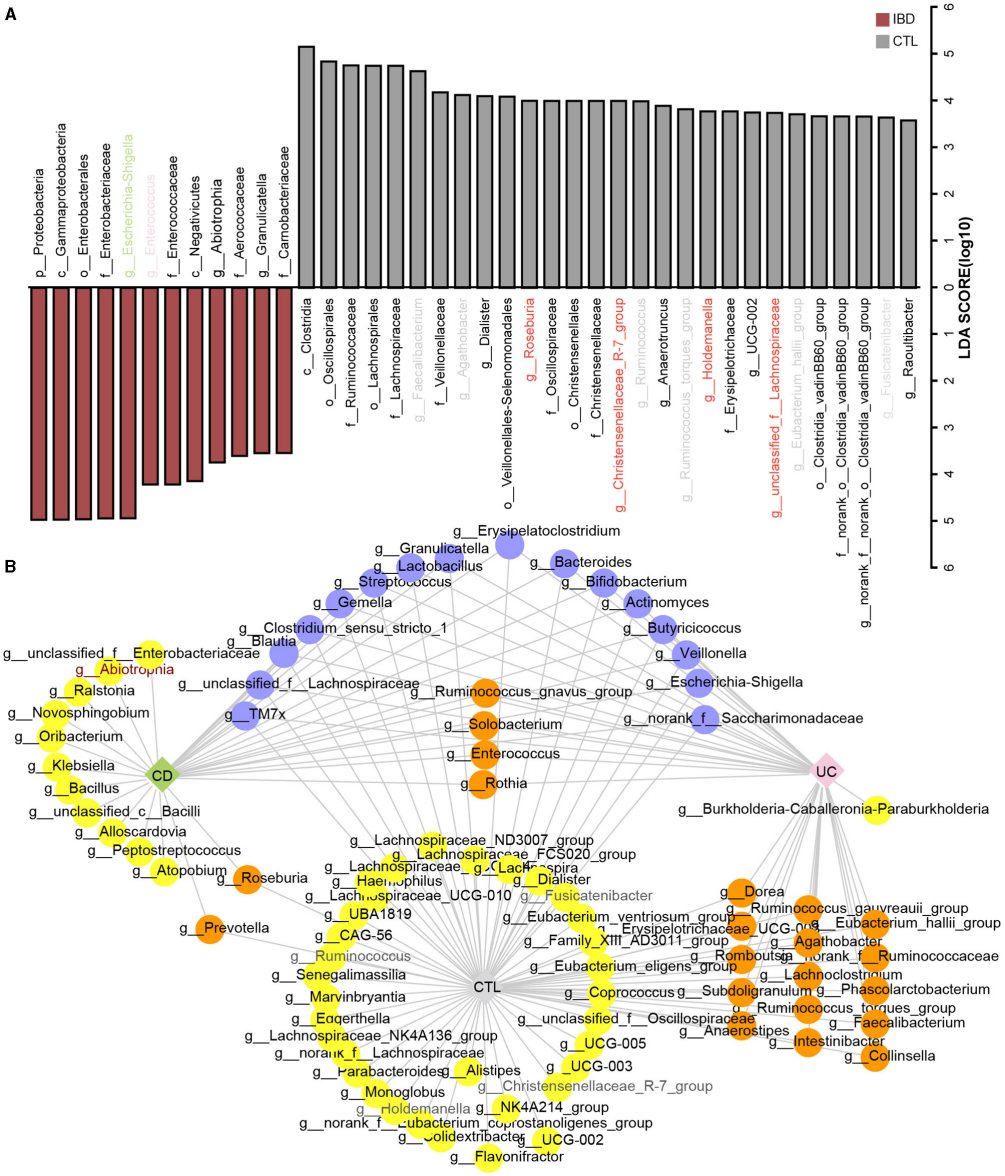
The taxonomy biomarkers across different groups. **(A)** The results of LEfSe analysis in the 16S rRNA profile highlight distinct microbial markers between IBD and CTL. The specific bacterial genera are colored with the same color as **(A)**, while red color means both identified in CD and UC when paired with healthy participants. The threshold of the LDA score is 3.5, and it is the same with Supplementary Figures 2A, B. **(B)** The co-occurrence network is derived from 16S rRNA sequencing. In this network, diamonds denote different group classifications, whereas green, pink, and gray correspond to CD, UC, and CTL, respectively, as indicated. Dots represent various bacterial genera, with color coding as follows: yellow represents bacteria unique to one group, orange represents bacteria associated with both CD and UC patients, and purple represents bacteria common across all three groups.

**Figure 4:**
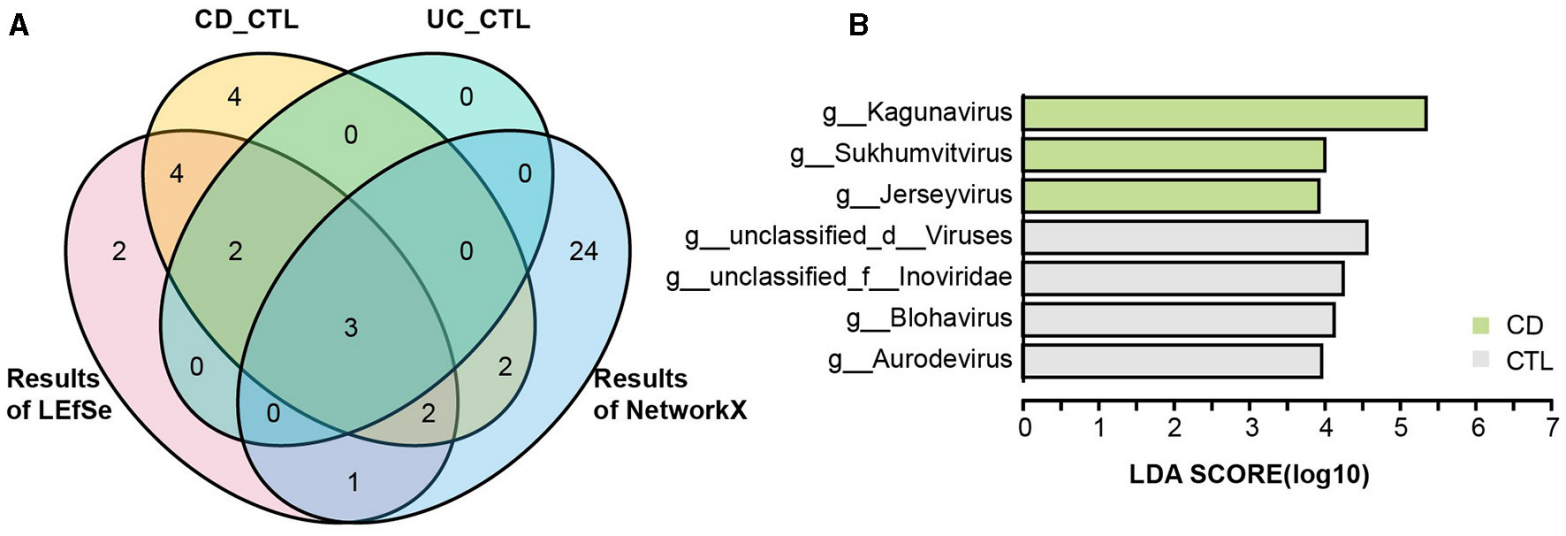
The taxonomy biomarkers of the CTL group and viral taxa. **(A)** A Venn diagram illustrates the distinct and shared significant genus-level microbes in the healthy participants. CD_CTL (yellow) and UC_CTL (turquoise) refer to the genera that have significantly increased in the healthy control participants paired with individuals affected by Crohn's disease (CD) and ulcerative colitis (UC), respectively, as shown in Supplementary Figure 2. The LEfSe results (red) indicate the genera that are significantly increased in healthy participants compared to those with inflammatory bowel disease (IBD), as presented in Figure 3A. The NetworkX results (blue) represent the specific microbial genera in the co-occurrence network of healthy participants, detailed in Figure 3B. **(B)** The LEfSe analysis results highlight differential viral taxa based on metagenomic sequencing profiles across groups. The LDA score is > 4, and the color scheme for this bar is consistent with Figure 1 to ensure consistency in visuals.

To identify the specific bacterial taxa (from phylum to genus level) among different groups, LEfSe analysis was applied in the 16S rRNA profile (Figure 3A). Proteobacteria at the phylum level and *Escherichia-Shigella, Enterococcus, Abiotrophia*, and *Granulicatella* at the genus level were significantly increased in IBD patients. To further explore the differences between CD and UC patients, we analyzed paired samples from each group, and the results showed that *Escherichia-Shigella* specifically increased in CD patients, while *Enterococcus* was specific to UC patients (Supplementary Figure 2A). *Faecalibacterium, Agathobacter, Roseburia, Christensenellaceae R-7 group, Ruminococcus, Ruminococcus torques group, Holdemanella, Eubacterium hallii group*, and *Fusicatenibacter* were specific bacterial taxa in the healthy control (CTL) group. *Roseburia, Christensenellaceae R-7 group*, and *Holdemanella* were identified in both CD and UC patients paired with healthy participants (Supplementary Figure 2B). The striking differences and interactions between bacterial communities were investigated by NetworkX (Figure 3B). *Abiotrophia* was specifically identified in CD patients, while *Enterococcus* co-occurred between CD and UC patients, and *Escherichia-Shigella* interactions were higher among the three groups. In the CTL group, *Christensenellaceae R-7 group, Fusicatenibacter, Dialister, Ruminococcus, Holdemanella, UCG-002*, and *Fusicatenibacter* were specifically identified. Furthermore, the *Christensenellaceae R-7 group, Fusicatenibacter*, and *Holdemanella* were identified as the core gut microbes in healthy participants by Venn analysis (Figure 4A). In addition, metagenome sequencing showed distinct viral profiles in IBD patients, including *Kagunavirus, Sukhumvitvirus*, and *Jerseyvirus*, that emerged as the predominant viral genera in CD patients, while our analysis did not reveal any specific viral signatures at the genus level in UC patients (Figure 4B). These results highlighted the divergent viral landscapes and dysbiosis of the gut microbiome in these two forms of IBD.

### 3.5 Functional analysis and virulence factor profiles based on metagenomics

**Figure 5:**
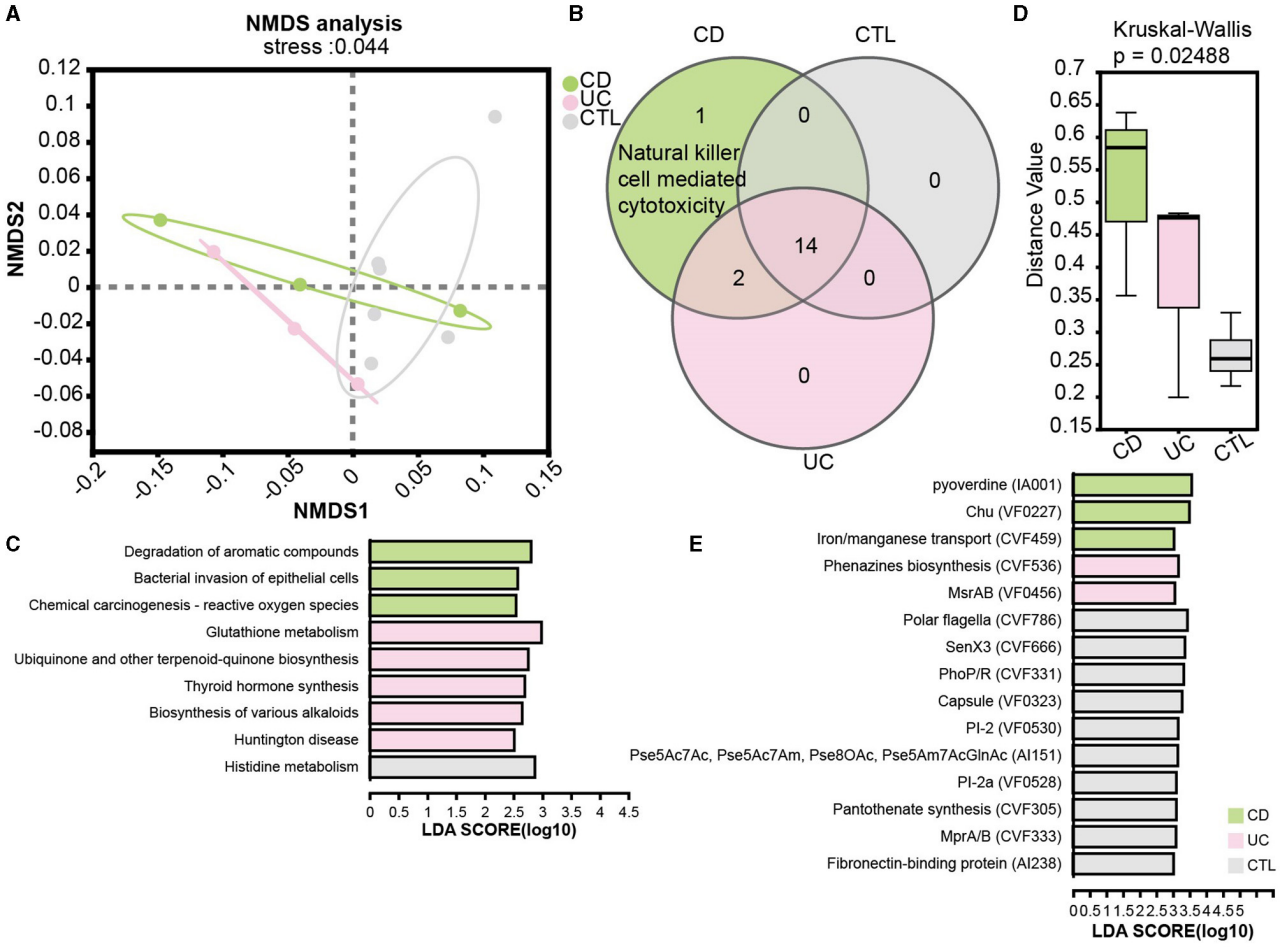
Functional analysis in patients and the significant virulence factors. **(A)** NMDS ordination plot based on KEGG orthologous group level 3. **(B)** A Venn plot for KEGG pathways specific in the immune system. **(C)** LEfSe analysis of KEGG functional predictions in human diseases. **(D)** The barplot of differences in community structure among different groups and Bray Curtis distance algorithm to calculate the distance value. **(E)** The LEfSe results showed significant virulence factors, LDA score is > 3. CD means Crohn's disease patients, UC means ulcerative colitis patients, and CTL means healthy participants in the metagenome profile.

To elucidate functional disparities, we employed the KEGG database in the metagenome profile. The NMDS analysis at the KEGG level 3 (stress = 0.044) revealed significant functional differentiation among the studied groups (Figure 5A). To focus on the influence of the immune system, the Venn diagram illustrated an enrichment of natural killer cell-mediated cytotoxicity in CD patients (Figure 5B). We then examined pathways specifically implicated in human diseases. Notably, the pathway bacterial invasion of epithelial cells and reactive oxygen species were prominently enriched in CD patients, while glutathione metabolism was enriched in UC patients (Figure 5C). Additionally, the virulence factor database (VFDB) was used to assess the virulence of unique target proteins, characterizing significant distance values across three groups (Figure 5D). Remarkably, phenazine biosynthesis, implicated in host tissue damage, and MsrAB, associated with oxidative stress resistance, emerged as unique virulence factors in UC patients. In contrast, virulence factors in CD patients appeared predominantly related to the uptake and utilization of essential minerals, which is crucial for bacterial survival and proliferation (Figure 5E).

**Figure 6:**
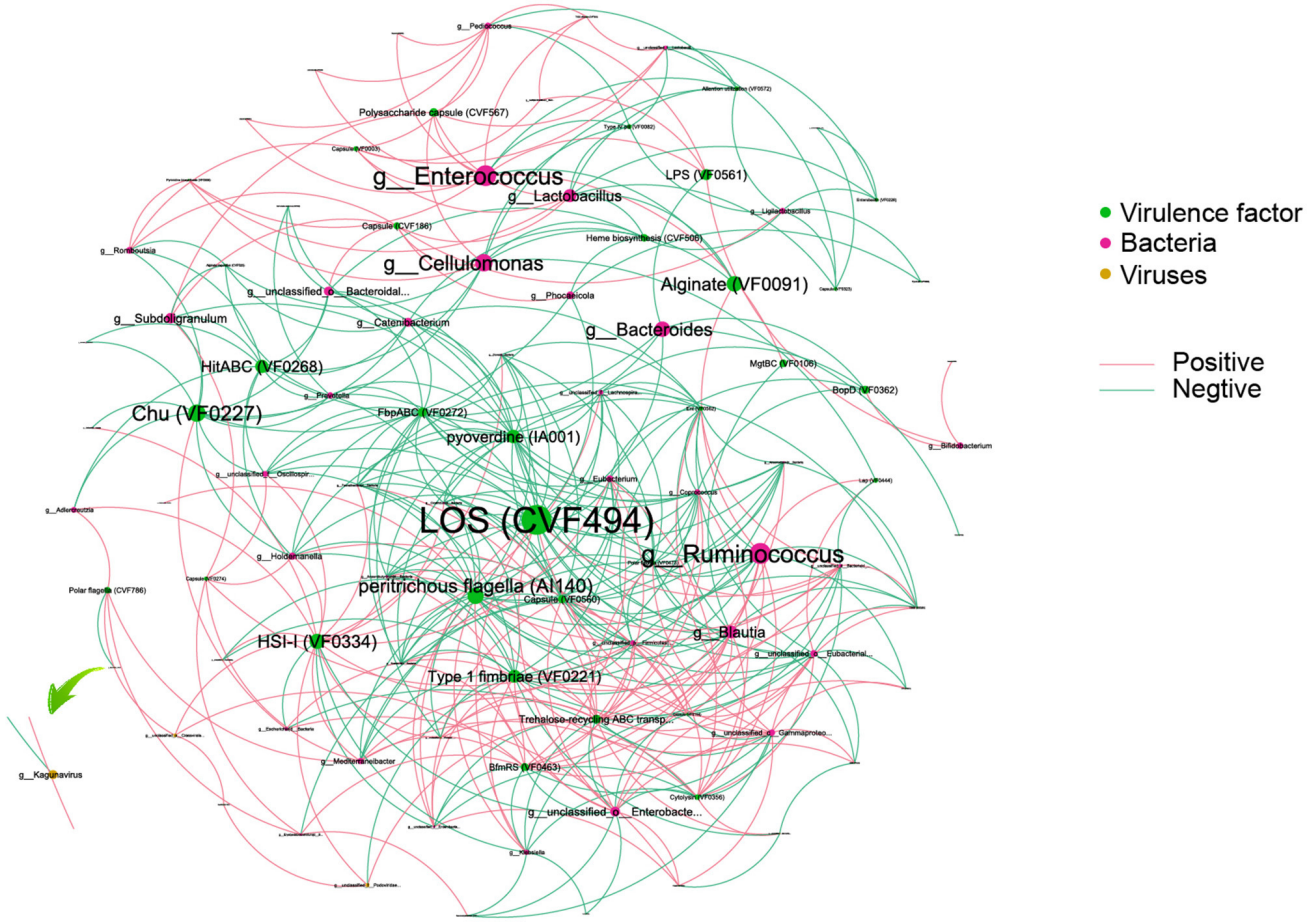
An interaction among bacteria, virus, and virulence factors. Different colors of dots represent the Bacteria (green), virulence factor (red), and viruses (yellow). The size of the dots represents the degree of interaction. The pink line represents a positive association, and the green line represents a negative association.

To reveal the complexity of microbial interactions within the gut microbiome, the Spearman correlation was performed between the microbiome at the genus level and various virulence factors (Figure 6). Lipooligosaccharide (LOS) (CVF494) demonstrated the most extensive and closest interactions with others. Chu (VF0227), which is specific to CD patients, showed interactions with bacteria, including *Escherichia* and *Subdoligranulum* genera. Within the bacterial community, *Enterococcus* and *Ruminococcus* exhibited the highest degree of interaction with virulence factors. Interestingly, our analysis revealed that only a limited number of viruses, six in total, showed interactions with virulence factors. Among these viruses, *Kagunavirus*, identified as a virus specific to CD patients, was a notable example of such interactions.

## 4 Discussion

In this case-control study, we investigated microbial dysbiosis in patients with IBD. Our findings demonstrate a marked decrease in both microbial richness and community diversity in these patients. Specifically, the Proteobacteria phylum was significantly more abundant in patients with CD. At the genus level, *Enterococcus* and *Escherichia-Shigella* were identified as biomarkers in IBD patients, with specific prevalence in UC and CD patients, respectively. In contrast, the *Christensenellaceae R-7 group, Fusicatenibacter*, and *Holdemanella* were more common in healthy controls. Functional analysis indicated distinct metabolic pathways between the two forms of IBD, pointing to the varied viral profiles in CD and UC patients. These results highlight the complex intricacies of microbial interactions within the gut microbiome.

Our findings reveal significant alterations in the gut microbiota of IBD patients and confirmed a notable decrease in microbial richness and diversity (Alam et al., 2020; Xu et al., 2022). A decrease in alpha diversity, critical for maintaining gut homeostasis, might compromise the mucosal barrier, thus impairing the gut's defensive mechanisms (Leibovitzh et al., 2022). Moreover, a noteworthy finding is the increased Proteobacteria phylum, highlighting its potential role in IBD pathogenesis (Mukhopadhya et al., 2012; Wiredu Ocansey et al., 2023) and suggesting a distinct microbial signature in these conditions. This observation is consistent with prior studies that reinforce the importance of Proteobacteria in CD patients (Vester-Andersen et al., 2019). In this study, no significant difference in microbial richness and diversity between CD and UC patients was identified, despite a previous study reporting inconsistent results regarding substantially lower species richness in CD patients compared to UC patients (Alam et al., 2020). Among newly diagnosed patients, alpha diversity showed no significant differences between UC and CD patients (Rausch et al., 2023), and the consistency in the microbial community at the early stages of the diseases might explain these inconsistent findings. These variations highlight the complex and multifaceted nature of microbial profiles in IBD.

The LEfSe analysis identified specific bacterial taxa associated with CD and UC patients, providing insights into the distinct microbial landscapes of these diseases. The decrease in commensal bacteria, such as the *Christensenellaceae R-7 group, Fusicatenibacter*, and *Holdemanella*, alongside an increase in *Enterococcus* and *Escherichia-Shigella*, suggests a progression toward a more pro-inflammatory gut environment. *Escherichia-Shigella* and *Enterococcus*, which are most prevalent in patients with IBD, may cause intestinal inflammation (Chen et al., 2014; Wiredu Ocansey et al., 2023). In an animal study, *Enterococcus* and *Escherichia-Shigella* were connected with a high-protein diet and negatively correlated with downregulated genes involved in innate immunity (Mu et al., 2016). The co-occurrence patterns of these two microbiomes might be related to the dietary patterns of participants. *Enterococcus* also stimulates apoptosis of intestinal epithelial cells deprived of the protective mucus layer (Golińska et al., 2013) and might contribute to the pathway of “bacterial invasion of epithelial cells.” Meanwhile, the *Christensenellaceae R-7 group* could be the cause of the increased *Escherichia-Shigella* levels and was also identified as a biomarker in the feces of healthy participants with high abundance (Cai et al., 2023). *Fusicatenibacter, Faecalibacterium*, and *Ruminococcus* are healthy gut-associated butyrate-producing bacteria known to be beneficial to host immunity (Maruyama et al., 2022; Malan-Müller et al., 2023), and butyrate is an important regulator that reduces mucosal inflammation and strengthens the epithelial defense barrier (Canani et al., 2011). Furthermore, *Holdemanella* was positively correlated with C18-3OH, a compound known for its anti-inflammatory properties (Pujo et al., 2021). *Abiotrophia* was previously shown to increase in healthy participants in a study in Saudi Arabia (Masoodi et al., 2020), which is inconsistent with our study. The genus *Abiotrophia* consists only of *Abiotrophia defectiva* and is related to a higher incidence of endocarditis among IBD patients (Wong et al., 2017; Bhardwaj et al., 2018), indicating a potential role in modulating gut inflammation. These alterations in microbial composition and diversity could potentially contribute to the pathogenesis and clinical manifestations of IBD. Moreover, the core biomarkers identified are consistent across different ethnic groups, indicating that the microbial signatures linked to IBD may possess a degree of universality.

Functional analysis suggests different pathogenic mechanisms. A previous study also revealed that CD patients showed a 3-fold higher level of NK activity than that detected in UC patients (Van Tol et al., 1992). Additionally, the enrichment of reactive oxygen species in CD patients mediates the activation of NK cells (Sun et al., 2020). On the other hand, glutathione metabolism was also significantly enriched in IBD patients (Santoru et al., 2017). The oxidized glutathione content in the mucosa displayed a significant positive correlation with both clinical and histological indicators of disease severity among patients with UC (Iantomasi et al., 1994). Furthermore, the potential causality of oxidative stress and the underlying biological mechanisms in CD was identified (Xu et al., 2023). This finding suggests that, while oxidative stress is implicated in both UC and CD patients, its role may differ slightly between the two conditions.

The analysis of the viral profile revealed a notable trend where viruses were significantly more abundant in CD patients than in UC patients, with three specific viruses showing a marked increase in CD patients. This observation, which is inconsistent with previous findings (Zuo et al., 2019), may be due to variability in the mucosal virome, potentially influenced by differences in sampling sites or techniques. Moreover, a significant relationship between *Kagunavirus* and polar flagella (CVF786), with a notable increase in healthy participants, was identified (*R* = −0.71, *p* = 0.01). The surface components of flagella, including pili and capsular polysaccharides, act as microbial-associated molecular patterns that regulate a cellular protease-dependent signaling cascade. This cascade produces various cytokines and chemokines, alleviating inflammation and enhancing intestinal epithelial function (Liu et al., 2020), and potentially enables beneficial bacteria to colonize niche environments within the gut. This negative correlation suggests that the presence of the virus might inhibit the motility and colonization capabilities of healthy bacteria in CD patients. This pattern highlights the complexities of host-microbe interactions and suggests *Kagunavirus* as a potential disease marker, warranting further investigation into its mechanisms.

The analysis of the interaction between the microbiome and the virulence factor revealed that the majority of linkages persisted between bacteria and virulence factors, with the *Enterococcus* genus appearing to have a substantial impact on inflammatory processes (Golińska et al., 2013). A notable positive correlation (*R* = 0.61, *p* = 0.036) was identified between the *Escherichia* genus and Chu (VF0227), a heme permease protein. This relationship held particular importance due to the role of Chu (VF0227) in iron acquisition and utilization, potentially facilitating the proliferation of adherent-invasive *Escherichia coli* within the inflamed intestines of human hosts (Abdelhalim et al., 2020). This positive regulatory effect might drive the colonization of adherent-invasive *Escherichia coli*, exacerbating intestinal inflammation. The interaction between *Escherichia coli* and Chu (VF0227) highlighted a crucial element of microbial survival and virulence within the host environment.

In summary, our study offered comprehensive insights into the altered microbial composition, interactions, and functionalities in IBD patients, with the case-control approach controlling for potential confounders. These findings significantly enhanced our understanding of the relationship between the gut microbiome and IBD pathogenesis. Additionally, the main biomarkers identified were consistent across various ethnic groups, indicating that the microbial signatures associated with IBD might exhibit a degree of universality, transcending diverse geographical backgrounds. However, this study has certain limitations, notably the need for a larger sample size to reinforce our findings. Future research could benefit from longitudinal studies to further elucidate the dynamic role of the gut microbiota in the pathogenesis process of IBD.

## Data availability statement

The raw sequence data reported in this paper have been deposited in the Genome Sequence Archive (Genomics, Proteomics & Bioinformatics 2021) in National Genomics Data Center (Nucleic Acids Res 2022), China National Center for Bioinformation/Beiing GSA: Institute of Genomics, Chinese Academy of Sciences (CRA015053).

## Ethics statement

The studies involving humans were approved by Ethics Committee of the Qinghai Provincial Traditional Chinese Medicine Hospital. The studies were conducted in accordance with the local legislation and institutional requirements. The participants provided their written informed consent to participate in this study.

## Author contributions

HW: Methodology, Supervision, Writing – original draft, Writing – review & editing, Data curation, Formal analysis, Visualization. YW: Data curation, Visualization, Writing – original draft, Writing – review & editing, Conceptualization, Investigation, Methodology, Project administration. LY: Data curation, Software, Visualization, Writing – review & editing, Investigation, Validation. JF: Data curation, Software, Writing – review & editing, Investigation, Methodology. ST: Data curation, Formal analysis, Software, Visualization, Writing – review & editing. LC: Data curation, Formal analysis, Visualization, Writing – review & editing, Investigation. WH: Data curation, Formal analysis, Investigation, Visualization, Writing – review & editing. JL: Data curation, Formal analysis, Investigation, Visualization, Writing – review & editing. XW: Investigation, Writing – review & editing, Writing – original draft, Conceptualization, Funding acquisition, Methodology, Project administration, Supervision.
