# Correction: Integrated 16S rRNA sequencing and metagenomics insights into microbial dysbiosis and distinct virulence factors in inflammatory bowel disease

**DOI:** 10.3389/fmicb.2026.1852209

**Published:** 2026-06-08

**Authors:** Haijing Wang, Yuanjun Wang, Libin Yang, Jiawen Feng, Shou Tian, Lingyan Chen, Wei Huang, Jia Liu, Xiaojin Wang

**Affiliations:** 1 Medical College of Qinghai University, Xining, China; 2 Qinghai University Affiliated Hospital, Xining, China; 3 Ningxia Hui Autonomous Region People's Hospital, Yinchuan, China; 4 Qinghai Provincial Traditional Chinese Medicine Hospital, Xining, China

**Keywords:** 16S rRNA, metagenome, inflammatory bowel disease, Crohn's disease, ulcerative colitis, case-control study

An incorrect number was provided for [National Science Foundation of Qinghai]. The correct number is [Grant No. 2020-ZJ-768].

The original version of this article has been updated.

