# Supplementary material for: Integrated 16S rRNA sequencing and metagenomics insights into microbial dysbiosis and distinct virulence factors in inflammatory bowel disease
Source: Front Microbiol. 2024 Mar 25;15:1375804. doi: 10.3389/fmicb.2024.1375804 (PMC10999624; doi:10.3389/fmicb.2024.1375804)
Supplement: Supplementary file 1 [file Data_Sheet_1.docx]

Supplementary Material


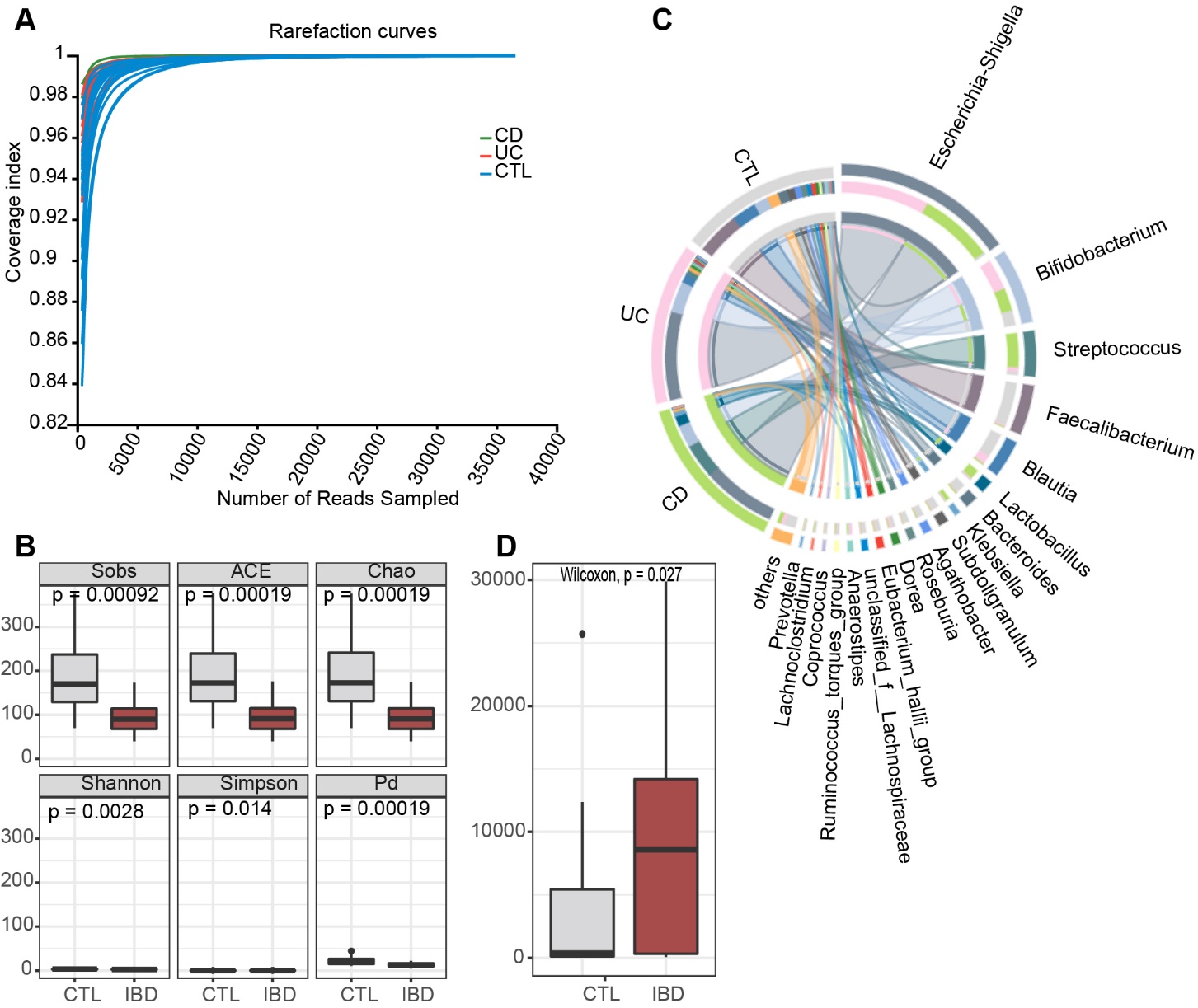
Supplementary Figure 1. Comparative analysis of microbial diversity and intercommunity relationships in different conditions. A: Rarefaction curves representing species richness estimation. B: The boxplot illustrate the difference in Alpha diversity between IBD patients and healthy participants, and a paired Wilcoxon test was conducted to assess statistical significance. C: Circos plot illustrating the microbial interconnection between three conditions at the genus level. D: The boxplot demonstrate the difference in phylum Proteobacteria between IBD patients and healthy participants, with a paired Wilcoxon test being conducted for statistical analysis.


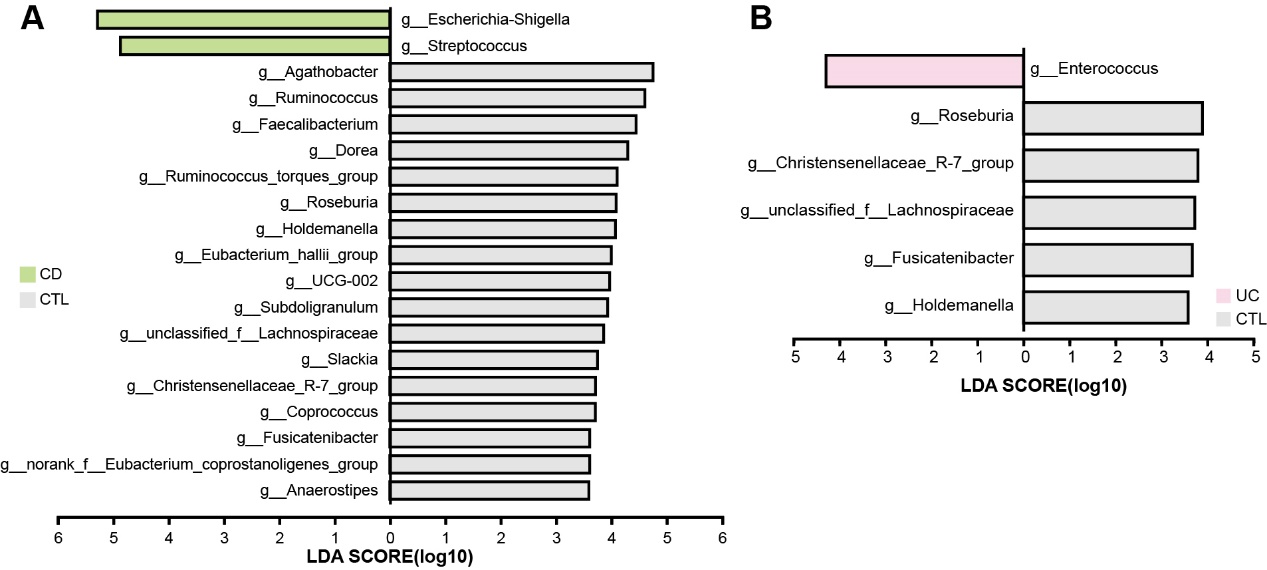


**Supplementary Figure 2.** Differential abundance of microbial taxa in disease conditions and control. A: LEfSe results indicating the taxa with a statistically significant difference in abundance between CD patients and healthy participants (CTL). B: LEfSe results displaying the taxa with a statistically significant difference in abundance between UC patients and healthy participants (CTL).
